# Optimized Sharable-Slot Allocation Using Multiple Channels to Reduce Data-Gathering Delay in Wireless Sensor Networks

**DOI:** 10.3390/s16040505

**Published:** 2016-04-09

**Authors:** Phan Van Vinh, Hoon Oh

**Affiliations:** 1 School of Engineering, Eastern International University (EIU), Binh Duong City 822096, Vietnam; vinh.phan@eiu.edu.vn; 2 Ubicom Lab, School of Computer Engineering and Information Technology, University of Ulsan, P.O. Box 18, Ulsan 680-749, Korea

**Keywords:** slot scheduling, optimized sharable slot, multi-channel, reliability, gathering delay

## Abstract

The demand for event-driven real-time applications for timely and reliable data acquisition is growing in industrial sectors. However, it is challenging to satisfy the requirements since constraints such as limited available energy and bandwidth are inherent in a wireless sensor network. To deal with timely delivery, one desirable approach is to improve network throughput so that more real-time applications with tighter time constraints can be satisfied in any given network. To deal with reliable delivery, the use of a carrier sense multiple access mechanism for data transmission is preferred, along with the use of a sharable slot within which multiple nodes compete to send data. Thus, we present a method of using multiple channels and a way to optimize the size of the sharable slot. The proposed channel-slot–scheduling algorithm tries to optimize the size of a sharable slot when multiple channels are used. The algorithm also deals with situations where nodes generate multiple data packets in each round of a data-gathering period. It is shown through simulation that our approach greatly outperforms others on some selected metrics.

## 1. Introduction

Recently, demand has been growing for wireless sensor networks (WSNs) in industrial sectors where a large number of sensor nodes cooperate to perform data gathering for a device or process control as well as for traditional data monitoring. One example of an industrial WSN application is the safety monitoring and control system (SMOCS) [1], which is used to ensure safety in a working environment by collecting and analyzing data readings (or context information) from the monitored objects. Based on the context of the collected information, the SMOCS server needs to take the appropriate actions. For example, the server may require some specific data (*i.e.*, multimedia data) with high traffic rate from specific sensors embedded with audio/video camera devices for better judgment of the situation. Therefore, with different traffic demands in sensor nodes, there is a need for deterministic data acquisition times to provide real-time services more effectively. However, it is a challenge to shorten the data-gathering time of applications with varying traffic in wireless multi-hop sensor networks since a node's bandwidth demand may vary over time, depending on a particular situation or application.

For data transmission in WSNs, most of the existing media access control (MAC) protocols use either carrier sense multiple access (CSMA) or time division multiple access (TDMA). In fact, contention-based MAC protocols that use CSMA usually try to send data with their best effort, and are thus very flexible in low-data-rate applications [2,3,4,5]. However, when traffic load increases, performance can be greatly reduced due to an increasing number of collisions and retransmissions. On the other hand, some TDMA-based MAC protocols usually allocate slots to nodes so that data transmission can be performed in a contention-free manner [6,7,8,9], making them more suitable to high-data-rate applications. However, if the allocated slots do not correctly satisfy the demand of the nodes, either some time slots are wasted if they exceed the demand or some data might be missed if the slots are not sufficient. Therefore, it is highly desirable to find the optimized length of a slot (and thus, total data gathering time), considering varying traffic load conditions.

Due to the limited energy resources, contention-based approaches that use a single channel for transmission usually focus on enhancing energy efficiency for low duty-cycle applications by reducing idle listening as much as possible. T-MAC [3] with an adaptive duty-cycle tries to mitigate the wasted time in S-MAC [2] by allowing a node to sleep earlier if there is no data to transmit or receive within a specific period of time. By employing a low-power listening technique, B-MAC [4] can reduce waiting time in channel access, thereby minimizing energy consumption. Since contention-based MAC protocols usually suffer from data collisions and long delays when the traffic load is high, contention-free MAC protocols try to solve the problems by allocating slots to nodes so they can send data in a contention-free manner. GinMAC [6] introduced slot scheduling to enhance the reliability of data transmission; however, its design is targeted to fixed, small, tree-based networks. TDGEE [7] builds an energy-balanced tree where sensor nodes can send data with different transmission power levels. IH-MAC [8] tries to improve energy efficiency by integrating broadcast scheduling and link scheduling. In RT-MAC [9], nodes four hops away are allowed to send data at the same time to mitigate interference and to enhance throughput. In TreeMAC [10], nodes configured vertically in a tree can reuse the same time slot to reduce the schedule length, which may cause irregular interference. To avoid this problem, instead of invoking slot reuse, I-MAC [1] allocates the dedicated slots to sensor nodes, according to demand, in a way such that all receiving slots get priority over any sending nodes, thereby maximizing data aggregation and filtering and reducing energy consumption. However, I-MAC may not be suitable for large-scale networks, because the data-gathering time tends to increase significantly in accordance with an increase in tree depth.

Another approach to handling varying traffic loads and reducing data-gathering time is to use a multi-channel technique [11,12,13,14,15,16,17]. In this context, MMSN [11] is the first multi-channel approach, especially designed for WSNs, that uses frequency assignment and a contention-based mechanism for data transmission within a time slot, resulting in high contention and long delays in dense networks. MC-LMAC [12] aims at maximizing network throughput by exploiting a multi-channel technique. However, the clash problem may affect its performance when multiple senders transmit data to the same receiver within the same time slot. Y-MAC [13] uses a channel-hopping technique and contention-based channel access for transmission. However, it is not easy to determine a suitable length for the schedule, which significantly affects delivery latency. TFMAC [14] exploits a multi-channel technique to improve throughput. To provide a conflict-free schedule, each node can use different channels within different time slots for data transmission. However, channel coordination between nodes may incur high costs, especially in dense networks. In TMCP [15], to reduce the degree of contention among neighboring nodes and to increase network throughput, a tree network is divided into multiple small networks that have the same root and one dedicated channel for communications. Unlike other existing approaches, there is no control channel in EM-MAC [16], and thus, it allows each node to dynamically select a channel and wake-up time by using a pseudo-random function.

Wireless HART and ISA100 [18], as industrial standards, exploit a slotted frequency-hopping technique to enhance transmission reliability. However, there is a need to design a scheduling mechanism to achieve high performance in data gathering. Thus, some studies [19,20] were conducted into efficient link scheduling in WirelessHART. In fact, Saifullah *et al.* introduced real-time transmission scheduling based on a branch-and-bound technique [19], as well as an efficient algorithm to cope with the network dynamics in WirelessHART networks. Han *et al.* presented reliable routing graphs that address the reliability problems in routing [20], as defined in the WirelessHART standard, to achieve end-to-end real-time transmission. DICSA [21] is a distributed link-scheduling approach based on network layer information, and uses a child-parent relationship to perform slot assignment. Thus, it can provide concurrent and collision-free slot reservation while significantly reducing overhead. The work by Dezfouli *et al.* [22] tries to address the problem of real-time communications in dynamic WirelessHART networks by introducing an efficient solution that can support the mobility of sensor nodes. However, this is still one of the critical issues in WSNs, and at present, there is no complete solution that can achieve a level of performance that can satisfy the service scenarios of real industrial applications.

The current IEEE 802.15.4 MAC has some problems in supporting low-power WSNs, *i.e.*, high energy consumption, and interference or fading problems. To cope with these problems, the IEEE 802.15.4e standard combines TDMA and a channel-hopping technique (similar to the HART technology) [23]. However, it does not address, in a concrete manner, how a link schedule is made. Thus, some schedule-based approaches associated with this standard were recently presented [24,25,26]. These approaches use either centralized scheduling [24], in which a schedule is made by a centralized node (sink or gateway), or distributed scheduling [25,26], in which the scheduling pattern is built based on topology information at individual node. Since they usually require all information about the network topology, it is hard to adapt to the changes in traffic load quickly.

It is worth mentioning that the aforementioned approaches do not address data aggregation during convergecast. That means each generated packet is usually individually sent to a server, without aggregation, referred to as a *raw-data gathering*. For example, some researchers [27,28,29] tried to find an optimal schedule length for convergecast data transmission. Due to the use of a dedicated slot with the same size, part of the time slot might be wasted if each node has different amounts of data to transmit. On the other hand, the generated packets can be aggregated at the receiver before forwarding to the sink, referred to as *aggregated-data gathering*. This way of data collection is designed to reduce network overhead and energy consumption accordingly. Therefore, some approaches [30,31,32] are aimed at finding optimal contention-free scheduling with the use of a multi-channel technique in an aggregation tree such that the aggregated data can reach the sink in minimal time.

In this study, we consider a protocol that allocates a sharable slot to each level of a tree, and the nodes at the same level compete to transmit their data within the sharable slot [33]. This way of scheduling maximizes data aggregation. However, it is hard to effectively determine the sharable slot size. Therefore, we propose an optimized sharable slot-scheduling algorithm that tries to optimize the size of a sharable slot with the use of multiple channels, taking into consideration changes in traffic load. Every node determines its own exact bandwidth demand, which accounts for an optimal size of a sharable slot, by collecting the child’s bandwidth demand and by calculating the total number of packets that need to be forwarded in each round of the data-gathering period. Moreover, multiple channels are utilized to enhance concurrent data transmission. A pseudo-random function [34] is employed to efficiently assign different channels to different siblings at the same tree level. In this way, channel competition for data transmission can be reduced significantly.

The rest of this paper is organized as follows: Section 2 gives preliminary information about this work. In Section 3, we present the formal design of our approach in detail. Section 4 describes the simulation setup and the simulation results. Finally, we provide conclusions and suggest future work in Section 5.

## 2. Preliminary

### 2.1. Network Model

**Figure 1:**
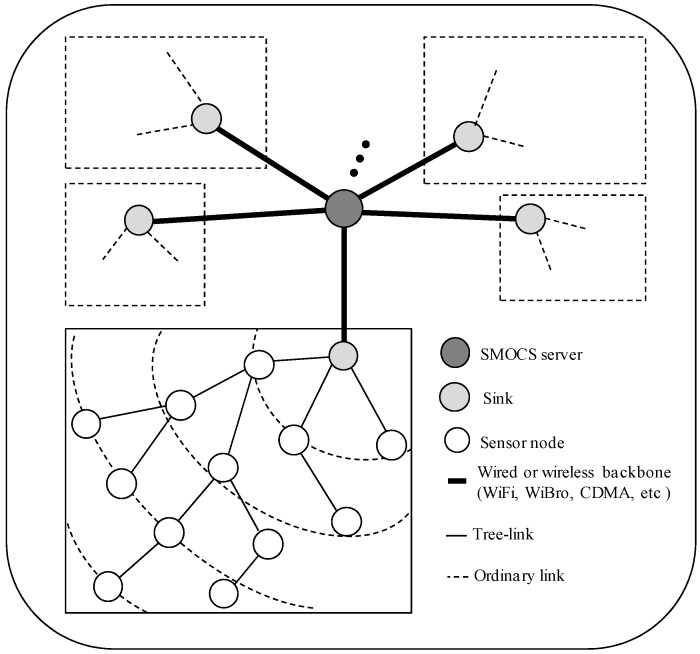
An illustration of a network model for a SMOCS system.

Figure 1 illustrates a network model that is especially designed for the SMOCS system [1]. There are *m* available channels for data communications. It is assumed that transmissions on different channels can be carried out simultaneously without interference, and each sensor node is embedded with only one half-duplex transceiver for communications.

Two communicating nodes can have a link if they are located within transmission range of each other. If a node joins a tree, it becomes a tree-node; otherwise, it is an orphan. The link between a node and its child is called a tree-link. This link can be affected by node failure, battery drainage, or environment factors.

In this approach, the network operates in repeated cycles. A cycle is organized into multiple sharable slots, and the nodes located at the same level (the same distance in hops to a sink) in the tree topology use the same sharable slot for data transmission. Therefore, the number of sharable slots corresponds to the maximum tree size (the highest level of the tree). A node can wake up at the assigned slot to transmit or receive data and enter a sleep mode to preserve energy at other times. Within one cycle, each node can create multiple data packets and forward them to a server. Therefore, the design of a communication protocol should adapt to various traffic loads to achieve desirable performance.

### 2.2. Notations

For the rest of the paper, the following notations are used for convenience:

- *T*(*i*): A set of nodes that belongs to a tree rooted at node *i*
- *n*(*l*): A set of nodes at level *l*
- *S*(*i*): A sibling group of node *i*
- *S*(*i*)′: n(*l*) − S(*i*)
- *C*(*i*): Child list for node *i*
- *p*(*i*): Parent of node *i*
- *level*(*i*) or *l_i_*: Level of node *i*
- η(*i*): Number of packets that node *i* generates during one round of data acquisition
- *SS*(*l*): Size of sharable slot for nodes at level *l*
- *SS^Rx^*(*i*) = *SS^Rx^*(*level*(*i*)): Size of sharable slot of node *i* for receiving data (also indicates receiving sharable slot allocated to the level to which node *i* belongs)
- *SS^Tx^*(*i*) = *SS^Tx^*(*level*(*i*)): Size of sharable slot of node *i* for sending data (also indicates sending sharable slot allocated to the level to which node *i* belongs)
- *H*: Highest level of a given tree

### 2.3. Problem Identification

In the previously proposed sharable-slot approach [33], a sharable slot is allocated to each level of a tree, starting from the nodes at the highest level down to those at the lowest, and thus data transmission is performed progressively from the bottom to the top of the tree through channel competition by nodes at each level. This approach constrains the nodes’ data transmission time by using a sharable slot, and ensures the reliability of hop-to-hop data transmission by employing a CSMA mechanism.

The sharable-slot approach targets real-time and reliable applications in industrial sectors where wireless links are often unstable due to various obstacles and interference unfriendly to wireless communications. However, it is very difficult to determine the optimal size of a sharable slot, because the size is affected by the number of competing nodes and by external interference from signals generated by other networks using the same frequency band. If a sharable slot allocated to a certain level is small, some of the nodes at that level may fail to transmit data. Conversely, if the size is big, some parts of a sharable slot may be wasted, resulting in a longer data-gathering time, or *superframe*. If the superframe gets larger, it may fail to support the real-time applications that need the tighter time constraints. Accordingly, it would be of great importance to optimize the size of a sharable slot.

Let us denote $SS^{Tx}\left(l\right)\;$as the time span taken for all nodes at level *l* to successfully execute data transmission using a specific protocol. Then, a superframe (*SF*) in the sharable slot approach can be expressed as follows:

(1) $$SF=\overset{H}{\underset{l=1}{\sum }}SS^{Tx}\left(l\right)\;$$

The problem of optimizing the *SF* can be reduced to optimizing the size of each sharable slot, that is, *SS^Tx^*(*l*), *l* = 1…H, as follows:

(2) $$SS^{Tx}\left(l\right)=\;\underset{i\in n\left(l\right)}{\sum }t_{Tx}\left(i\right)$$

where $t_{Tx}\left(i\right)$ denotes the maximum transmission delay of node *i* at level *l*. Then, $t_{Tx}\left(i\right)$ can be expressed in the form of the following function:

(3) $$t_{Tx}\left(i\right)=f\left(P,\left|S\left(i\right)\right|,\left|S{\left(i\right)}^{\prime }\right|,\text{nPkts}\left(i\right)\right)$$

where $t_{Tx}\left(i\right)$ depends on the used protocol, *P*; the number of siblings of node *i* is presented as |S(i)|; $\left|S{\left(i\right)}^{\prime }\right|$ is the number of non-siblings of node *i*; and the number of packets to be processed or forwarded by node *i* is denoted as *nPkts*(*i*).

**Figure 2:**
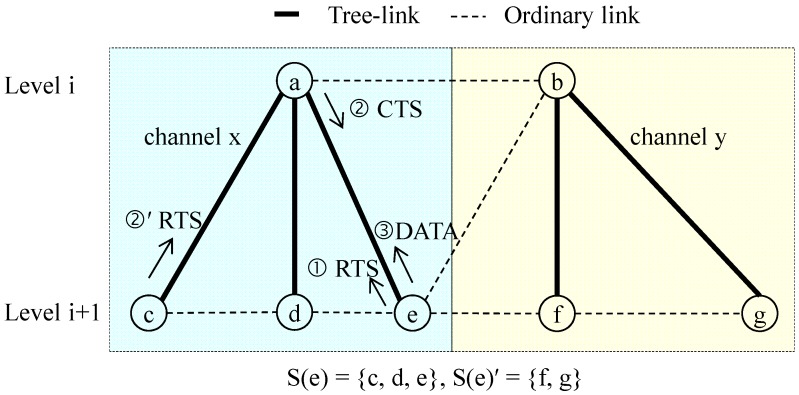
The use of multiple channels to reduce collision when using CSMA.

It is natural for more siblings to lead to more competition in data transmission. Therefore, if a CSMA mechanism is used for data transmission, we can reduce competition between the nodes by using multiple channels. As illustrated in Figure 2, if two channels *x* and *y* are used, sibling nodes *c*, *d*, and *e* compete for data transmission to node *a*. Furthermore, if one of the siblings, say *e*, initiates request to send (RTS) first by random competition delay, and if a hidden node, say *c*, does not initiate RTS until node *a* starts responding with clear to send (CTS), collision will not occur. Therefore, if a random delay is set in the multiple transmission times of RTS, we can avoid data collision.

If a dedicated channel is assigned to every sibling group, Equation (3) can be reduced to Equation (4) by removing $\left|S{\left(i\right)}^{\prime }\right|$:

(4) $$t_{Tx}\left(i\right)=f\left(P,\left|S\left(i\right)\right|,nPkts\left(i\right)\right)$$

Another benefit of using multiple channels is that the probability of parallel transmission can be increased significantly within a sharable slot, thereby increasing throughput. This can help satisfy more real-time applications with tighter deadlines. 

If every node generates multiple packets in each round of data collection, *nPkts*(*i*) can be expressed as follows:

(5) $$nPkts\left(i\right)=\underset{k\in T\left(i\right)}{\sum }\eta \left(k\right)$$

If η(.)=1, we get nPkts(i)=|T(i)|, where |*T*(*i*)| indicates the cardinality of *T*(*i*). 

If there is no competition, *nPkts*(*i*) has to be appropriate to the number of slots that a node requires to forward data packets in its sub-tree, assuming that a slot indicates the time span that a node takes to transmit one packet without competition. 

In fact, the probability that the hidden terminal problem can occur within a sibling group can be negligible when CSMA is used, if the random delay is set properly, as explained in Figure 2. In that case, the packet transmission time of node *i* is independent of |*S*(*i*)|. Thus, Equation (4) can be changed to Equation (6):

(6) $$t_{Tx}\left(i\right)=f\left(P,nPkts\left(i\right)\right)$$

Equivalently, we get:

(7) $$t_{Tx}\left(i\right)=nPkts\left(i\right)\times onePacketDelay\left(i\right)$$

where *onePacketDelay*(*i*) corresponds to the time span that node *i* takes to transmit one packet to its parent. However, if collision is avoided within a sibling group, *onePacketDelay*(*i*) is the same for all nodes. It can be calculated easily by considering the data transmission process of RTS, CTS, DATA and ACK. Then, we can get an optimal sending sharable slot *SS^Tx^*(*l*) of level *l* as follows:

(8) $$SS^{Tx}\left(l\right)=max\left\{\sum_{j\in S\left(i\right)}t_{Tx}\left(j\right)\;|\;i\in n\left(l\right)\right\}\times onePacketDelay$$

where *onePacketDelay* is constant. Then, the problem of getting *SS^Tx^(l)* is simplified to the one of finding a sibling group with the maximum number of packets to transmit within a sharable slot, corresponding to the first term of Equation (8).

## 3. Protocol Design

### 3.1. Time Frame Structure 

**Figure 3:**
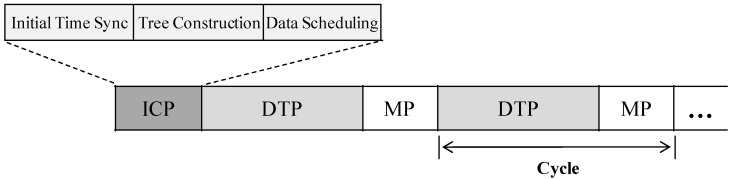
Time frame structure.

Figure 3 shows the time frame of our proposed approach, which starts with the initial construction period (*ICP*) followed by a repeating cycle consisting of the data transmission period (*DTP*) and the maintenance period (*MP*). At the beginning of the time frame (initial time synchronization), tree construction and data scheduling are executed. For time synchronization [35,36,37], flooding time synchronization protocol (FTSP) [36], which is proposed for tree-based topology networks, is used owing to its high accuracy from exploiting a linear regression technique. Next, data scheduling is performed to calculate bandwidth demands of the network, and then sharable data slots are assigned to the nodes at each level. The *DTP* is executed according to the scheduled data slots. In the *MP*, the operations (*i.e.*, time synchronization, tree maintenance, and scheduling recovery) are performed conditionally, if necessary.

### 3.2. Tree Construction

The construction of a tree is based on the principle that a node builds a bi-directionally reliable path, but takes the shortest path if there are two or more candidate parents that provide bi-directionally reliable links. If node *i* successfully sends data to node *j*, link (*i*, *j*) is said to be *reliable*. Accordingly, link (*i*, *j*) is said to be *bi-directionally reliable* (or *B-reliable*) if both link (*i*, *j*) and link (*j*, *i*) are *reliable*. If every tree-link in a tree is *B-reliable*, the tree is said to be a *B-reliable tree* [1].

In the beginning, every node enters a tree construction period to build a B-reliable tree topology for data communications. First, the sink starts the tree construction process by broadcasting a tree initiation (*T-Init*) message. When receiving *T-Init*, an orphan with a *B-reliable* link to the sink tries to join the tree by transmitting a tree join–request (*T-Req*) message. Upon receiving *T-Req*, the receiver sends a tree join–acknowledgement (*T-Ack*) message to the orphan node to confirm that it accepts the orphan as its child, a tree member. When the orphan receives *T-Ack*, it takes the source node as its parent. Note that a sender always attaches information about its neighbors with associated link quality accordingly, in the tree construction message, so that the receiving node can easily justify the quality of the appropriate link between them. Other nodes that overheard the *T-Req* message can invoke the same process to join the tree. If a node overhears multiple *T-Req* messages, it should join the member node at the lowest depth in the tree, as described in the shortest path algorithm [1]. 

### 3.3. Channel and Slot Scheduling

In this section, we formally describe three key things. First, we describe how a node synchronizes its channel with its parent’s channel, and how it takes a channel different from the channels of other sibling groups at the same level. Second, we present a method to determine the optimal size of a sharable slot when different sibling groups use different channels in a tree. Finally, slot scheduling is discussed.

#### 3.3.1. Distributed Channel Allocation

With the aim of mitigating overhead and enhancing flexibility, we use distributed channel scheduling in which a node can determine the channel on which to send or receive data without the need of channel coordination. For this purpose, a linear congruential generator (LCG) [34] is used to generate a random number with a given seed. This means that if we know the seed, we can determine the random sequence accordingly. Therefore, if the ID of each node is used as a *seed*, the output value when applying the LCG can be considered the channel index of that node. More specifically, the LCG can be expressed as:

(9) $$X_{n+1}=\left(aX_{n}+c\right)mod\;m\;(0\leq a,c<m;\;n\geq 0)$$

where *m* is a modulus, *a* is a multiplier, *c* is a increment, $X_{n}$ is the current seed ($X_{0}$ is the initial seed). If *m* is given as the number of available channels, and $X_{0}$ is set to the ID of a node, $\left\{X_{1},\;X_{2},\;\ldots ,\;X_{m-1},X_{m}\right\}$ presents the sequence of the channel index of the node. 

In our application, a node can use different channels to send and receive data. More specifically, a node uses its own ID as the seed to determine the receiving channel index while using its parent’s ID as the seed to determine the sending channel index for communications. This approach does not require any channel coordination between sender and receiver, therefore overhead is kept to a minimum.

#### 3.3.2. Bandwidth Demand Calculation

For convenience, the bandwidth demand of a node is simply expressed as the number of data packets that it has to forward since the transmission delay, *onePacketDelay*, of a packet is constant, as explained in Equation (8).

For a single channel, the bandwidth demand of a node consists of two factors: (a) the number of packets that the node generates and also receives from its children, and (b) the sum of the bandwidth demands that its children need. If each child uses a distinct channel, the children can receive packets from their own children in parallel; however, the children cannot transmit their packets in parallel, since they have the same parent as a receiver. Therefore, the sending-demand of the children is the total of the sending-demands of all children, whereas the receiving-demand of the children is given as the maximum of the receiving-demands of all children. More specifically, the bandwidth demand of node *i*, *B*(*i*), can be presented as follows:

(10) $$B\left(i\right)=\left(\sum_{k\in T\left(i\right)}\eta \left(k\right)+D\left(i\right)\right)$$

where *D*(*i*) is calculated by:

$$D\left(i\right)=\{\begin{array}{c} \left(\underset{x\in C\left(i\right)}{\sum }\underset{k\in T\left(x\right)}{\sum }\eta \left(k\right)\right)+max\left\{D\left(x\right)\;|x\in C\left(i\right)\right\}if\;C\left(i\right)\neq \emptyset \\ 0\;if\;C\left(i\right)=\emptyset \end{array}$$

If η(.)=1, Equation (10) is simplified as follows:

(11) B(i)=(|T(i)|+D(i))

where |*T*(*i*)| indicates the cardinality of *T*(*i*).

**Figure 4:**
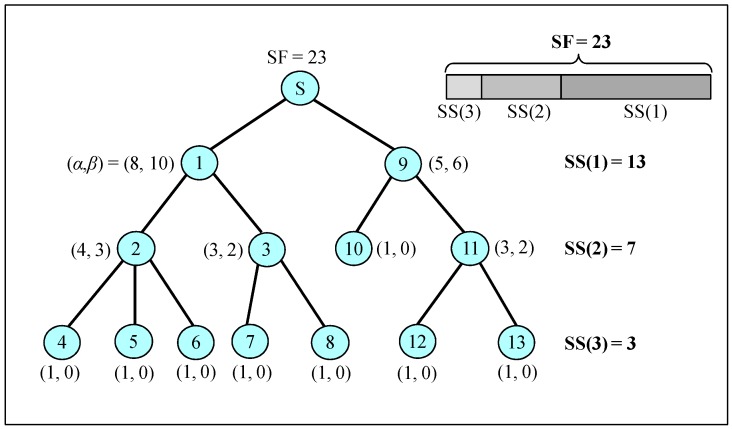
Bandwidth-demand calculation and the corresponding size of a sharable slot.

The bandwidth demand of each node can be counted by starting from the nodes at the bottom of the tree. Figure 4 shows an example with a simple tree of 14 nodes. The bandwidth demand calculated for each node is given in the figure as (α, β) with η(.)=1, where (α, β)=(|T(i)|,D(i)). Leaf node 4 has to send one packet, but does not have to give out bandwidth. Intermediate node 1 has two children: nodes 2 and 3. It has eight packets to transmit, resulting in (α = 8), and its two children, nodes 2 and 3, have sending demands of 4 and 3, respectively, and receiving demands of 3 and 2, respectively. Then, it takes the sum of sending demands and the maximum of the receiving demands, resulting in (β=10). 

#### 3.3.3. Sharable Slot Size

After collecting the bandwidth demands of all nodes, a sink produces a slot schedule that includes the size of each sharable slot and its starting time. The size of a sharable slot is determined by a sibling group that has the maximum number of packets to transmit, because different sibling groups can transmit packets in parallel. Therefore, the size of a sharable slot, *SS*(*l*), for nodes at level *l*, can be calculated by:

(12) $$SS\left(l\right)=max\left\{\sum_{x\in S\left(i\right)}\sum_{k\in T\left(x\right)}\eta \left(k\right)|\;i\in n\left(l\right)\right\}=max\left\{\left(\sum_{k\in T\left(p\left(i\right)\right)}\eta \left(k\right)\right)-\eta \left(p\left(i\right)\right)|\;i\in n\left(l\right)\right\}$$

If every node generates one packet in each round of data acquisition, that means η(.)=1, and Equation (12) can be rewritten as follows:

(13) $$SS\left(l\right)=max\left\{\sum_{x\in S\left(i\right)}\left|T\left(x\right)\right|\;|\;i\in n\left(l\right)\right\}=max\left\{\left(\left|T\left(p\left(i\right)\right)\right|-1\right)\;|\;i\in n\left(l\right)\right\}$$

This corresponds to the sum of α’s for the sibling group that has the maximum number of packets to transmit, as illustrated in Figure 4. For example, at level 2, S(2) = S(3) = {2, 3} has seven packets, and S(10) = S(11) = {10, 11} has four packets at level 2; therefore, *SS*(2) = 7.

#### 3.3.4. Slot Scheduling

**Figure 5:**
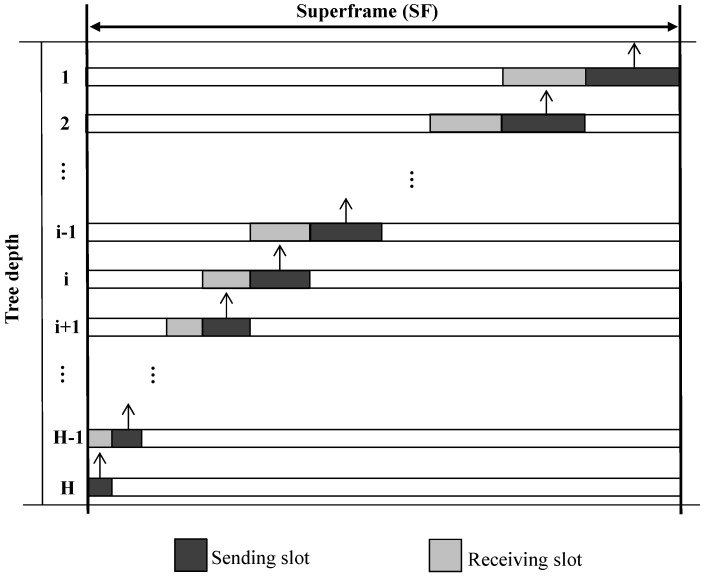
An illustration of a slot scheduling.

To maximize data aggregation and filtering, sharable slots need to be scheduled, such that data transmission is performed progressively from the highest level to the lowest in a tree. Thus, the highest level is assigned a sharable slot first, and then the lower one is assigned next, and so on, as illustrated in Figure 5.

As soon as ICP finishes, slot scheduling is performed starting from the sink. In every cycle, a node at each level determines the starting slot time for receiving data from its children, *StartRxSlot*, and for sending data, *StartTxSlot*, as follows:

(14) $$StartRxSlot_{t}\left(l\right)=StartCycle\left(t\right)+\sum_{k=l+2}^{H}SS\left(k\right)$$

(15) $$StartTxSlot_{t}\left(l\right)=StartCycle\left(t\right)+\sum_{k=l+1}^{H}SS\left(k\right)=StartRxSlot_{t}\left(l\right)+SS\left(l+1\right)$$

where *StartCycle*(.) indicates the starting time of a cycle.

Accordingly, a node at level *l* can start receiving data packets from its children at $StartRxSlot_{t}\left(l\right)$ computed by Equation (14) and can start transmitting data to its parent at $StartTxSlot_{t}\left(l\right)$ computed by Equation (15). 

### 3.4. Data Transmission and Recovery

#### 3.4.1. Data Transmission

According the slot scheduling, a sharable slot is allocated to each level of a tree, starting from the nodes at the highest level down to those at the lowest one, therefore data transmission is performed in the level-by-level fashion. As a result, within a particular slot, only the nodes at the corresponding level is allowed to transmit data, thus the channel contention is limited to the nodes at the same level. However, within a sharable slot, the nodes at the same level have to contend for transmission, which may result in collision. To solve this problem, two methods can be applied: (*i*) for the nodes within the same sibling group, we employ the CSMA scheme for data transmission; (*ii*) to solve the interference problem by the nodes belonging two different sibling groups, a dedicated channel is allocated to each parent node to receive data (which is done by the distributed channel allocation scheme presented in Section 3.3.1).

**Figure 6:**
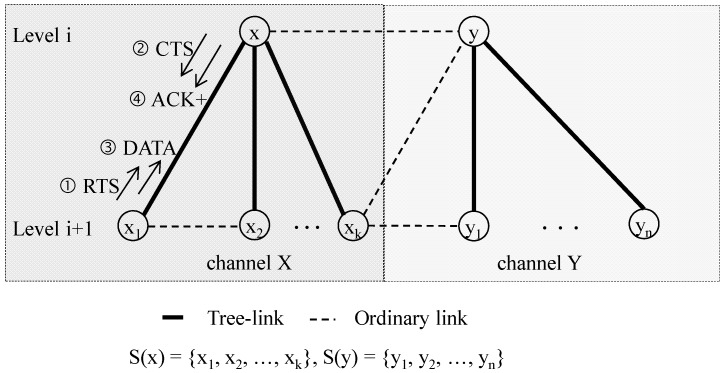
Data transmission sequence within a sharable slot.

As discussed above, channel contention can be limited to a group of sibling nodes in our approach. Therefore, every node competes with its siblings for data transmission within a sharable slot, as illustrated in Figure 6. Suppose that a node *x_1_* with its parent *x* has *k* − 1 siblings, represented as *S*(*x_1_*) = {*x_1_, x_2_, …, x_k_*}. Then, the *k* number of nodes will compete with each other to contend for the same channel (channel *X*, for example).

If *SS^Rx^*(*i*) starts, every node in *S*(*x_1_*) generates a random delay number in delay window *DW*[0, D]. A node where random delay number times *delay_slot* expires will send RTS to its parent *x*. In this case, *delay_slot* indicates the time span required to transmit one control packet. If two or more nodes generate the same random number, obviously, collision will occur at receiver *x*. In this case, they generate a random delay number again with a doubled delay window *DW*[0, 2D].

If a certain node receives CTS, it starts transmitting DATA immediately. When parent *x* receives DATA successfully, it responds with *ACK+*, which is a modified ACK to include a *spare time* (see the following subsection). As soon as a node succeeds in data transmission, it goes into sleep mode until the corresponding sharable slot of the next superframe starts.

#### 3.4.2. Spare Time Utilization

In industrial sensor networks, a wireless communications link can be broken due to fading, shadowing, intervention of obstacles in the industrial field, or battery depletion, *etc.* Thus, a MAC protocol should be able to respond effectively to the dynamic change in topology. In this work, we devise *a spare time utilization scheme* to provide a chance to transmit buffered data to nodes that have lost their parents. The *spare time utilization* *scheme* consists of two processes: *spare time announcement* and *spare time utilization*.

##### (a) Spare time announcement

A sharable slot is determined based on a sibling group with the maximum number of packets among the sibling groups. Thus, the sibling groups may not consume a sharable slot completely, thus producing some spare time. Furthermore, the filtering and aggregation of packets and the loss of packets in data transmission will additionally produce spare time. Therefore, the consumed time of node *i* within the sharable slot, *CT*(*i*), in the special case of η(.)= 1, has the following constraint:

(16) $$CT\left(i\right)\leq \;SS^{Rx}\left(i\right)=max\left\{\left(\left|T\left(k\right)\right|-1\right)\;|\;k\in n\left(l_{i}\right)\right\}\;$$

Then, spare time *ST*(*i*) is presented as follows:

(17) $$ST\left(i\right)=SS^{Rx}\left(i\right)-CT\left(i\right)$$

Note that *SS^Rx^*(*i*) = *SS^Rx^*(*l*), since every node at the same level *l* uses the same sharable slot, and then *ST*(*i*) for η(i)≠1 can similarly be calculated.

An orphan that lost its parent has to remain active to find an opportunity to send data or rejoin the network. In that case, the orphan can utilize the spare time of its neighbor to salvage the data buffered in its queue. A spare time announcement uses a modified ACK message, *ACK+* as follows: *ACK*+(*i*) = (*level*(*i*), *ST*(*i*)). Upon receiving the last of the data from its children, a node computes *ST*(*i*) according to Equation (17) and broadcasts *ACK*+.

##### (b) Spare time utilization

**Figure 7:**
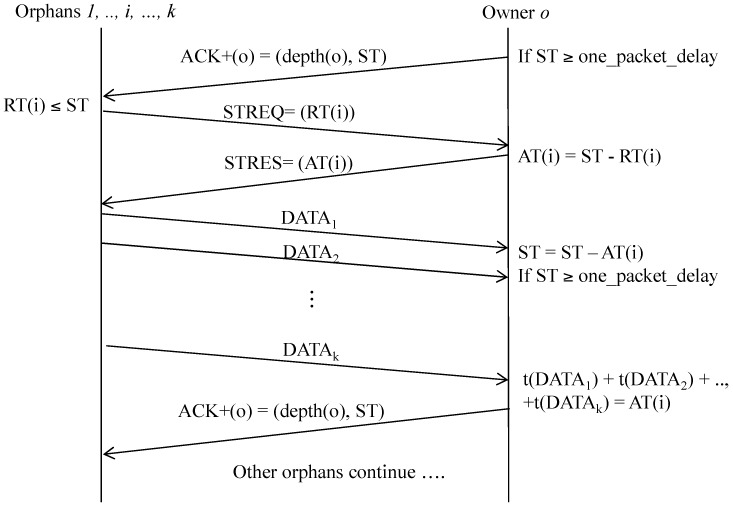
Spare time utilization process.

Upon receiving *ACK*+, an orphan sends a *spare time request* (*STREQ*) message to the spare time owner, *owner*, to request use of the spare time, with *STREQ*(i) = (*level*(*i*), *RT*(*i*)), where *RT*(i) indicates the *requested time* of node *i* and *RT*(i) < *ST*(*owner*), as illustrated in Figure 7.

Upon receiving *STREQ*, the owner replies with a *spare time response* (*STRES*) message that allows other nodes to use a part or all of the spare time, based on the remaining spare time. Specifically, *STRES*(owner) = (depth(owner), *AT*(owner)), where *AT*(owner) indicates the *allocated time* for requester *i*, and *AT*(owner) ≤ *RT*(i).

When the orphan receives *STRES* from the neighbor, it starts to send data to the neighbor. In this way, if a node has spare time to receive more data, it has to remain in the active state for a specified time after sending *ACK*+ to receive any data requests from its neighbors. Note that the size of the data (in time units) should not be more than the *ST* value.

### 3.5. One Packet Delay and Superframe Size

As discussed previously, *onePacketDelay* is calculated based on a *data transmission sequence*, RD-RTS-CTS-DATA-ACK+ where RD is the random integer value that a sender generates to avoid collision. The maximum of RD can be obtained from delay window *DW*[0, D] (in this paper, D is set to 5). If two or more nodes do not generate the same lowest random number, message collision will not occur. However, it is possible to experience collision even though the number of nodes in each sibling group is small. Thus, if collision occurs, a node generates a random number again after adjusting the delay window to DW[0, 2D] once and only once. If it fails again, it gives up sending its packet. Thus, the maximum random delay is limited to 2D.

Then, the upper bound of *onePacketDelay*, the maximum possible delay to transmit one packet, is given as follows:

(18) onePacketDelay≤2×D×delay_slot+t(RTS)+t(CTS)+t(DATA)+t(ACK+)

Considering the process of data transmission from one node to another, the transmission time of a data packet, say *x*, is given as follows:

(19) $$t\left(x\right)=t_{ts}\left(x\right)+t_{turnon}+t_{CCA}+t_{ppd}+t_{tx}\left(x\right)+t_{tr}\left(x\right)$$

where, $t_{ts}$ indicates the time to transfer a message from the MCU to a radio chip buffer, $t_{turnon}$ indicates the time to turn the radio chip on, $t_{CCA}$ indicates the time to perform a clear channel assessment (CCA), $t_{ppd}$ indicates the amount of time for the physical layer processing delay, $t_{tx}$ indicates the time to transmit or broadcast a message, and $t_{tr}$ indicates the time to transfer a message from the radio chip to the MCU at the receiver size.

In Equation (19), the values for $t_{turnon}$, $t_{CCA}$, and $t_{ppd}$ depend on the physical layer standard; however, the rest of them not only depend on the physical layer standard, but also on the size of the transmitted message. Based on the 802.15.4 standard with a transmission rate of 250 kbps (0.032 ms per byte) and a synchronization header, PHY header, MAC header, and footer of 11 bytes, we can calculate $t_{tx}\left(x\right)$ for message *x* as follows:

$$t_{tx}\left(x\right)=0.032\times \left(11+size\left(x\right)\right)\;\left(\text{in }ms\right)$$

Furthermore, $t_{CCA}$ = 0.128 ms and $t_{ppd}$ = 0.192 ms. We assume that $t_{ts}\left(x\right)$ = $t_{turnon}$ = $t_{tx}\left(x\right)$ ≅ 0. As a consequence, we get *t*(*X*), *X* ∈ {RTS, CTS, DATA, ACK+}, as follows:

t(X)=0.32+0.032×(11+size(X))(in ms)

Then, Equation (18) can be rewritten as follows:

onePacketDelay ≤2.688+2×D×delay_slot+0.032×(size(RTS)+size(CTS)+size(DATA)+size(ACK+)) (in ms)

Given that *delay_slot* is 0.832 (=t(RTS)) and size(RTS) = size(CTS) = size(ACK+) = 5 bytes, and size(DATA) = 100 bytes, we can get the lower bound and the upper bound of *onePacketDelay* when D is min value 0 and max value 5, respectively:

6.37 ms ≤onePacketDelay≤14.69 ms

Given the tree, we can calculate the size of an optimal sharable slot in packets for each tree level using Equation (12). In turn, we can get the optimal size of the SF in a packet. If *SF* corresponds to *k* *packets*, SF in seconds (*SFsec*), is expressed as follows:

k×6.37 ms ≤SFsec≤k×14.69 ms

This implies that a sink can at least collect data readings from all sensor nodes within k×14.69 ms.

## 4. Performance Evaluation

### 4.1. Simulation Setup

**Figure 8:**
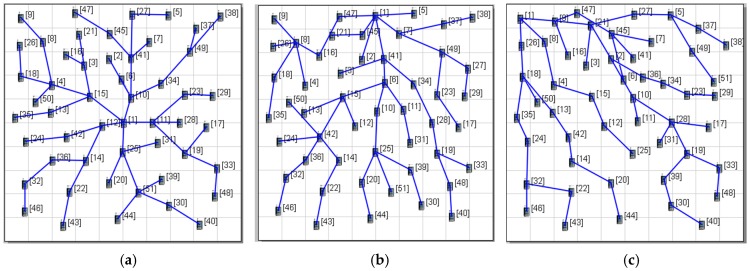
Simulation scenarios: (**a**) S1 with 50 nodes; (**b**) S2 with 50 nodes; (**c**) S3 with 50 nodes.

The performance of the proposed protocol (abbreviated as MCMAC) was extensively evaluated by using the QualNet 5.0.2 simulator [38]. In the network model, nodes are randomly deployed in an area of 100 × 100 (m^2^). Three scenarios, in which a sink is located at the center (S1), the top center (S2) and the corner (S3) of the deployment area were used to evaluate the effect of network length on performance, as shown in Figure 8 [17]. In the simulation, data transmission was also performed with consideration for the effects of the industrial environment (*i.e.*, path loss, shadowing, and multipath fading).

Since our target application is multi-hop WSN data gathering, it was assumed that one packet with a 32-byte payload length is generated at each source node in each round of data collection. The generated packets can be aggregated at the receiver before forwarding to the sink. The transmission of a packet may fail due to interference or the effect of the propagation environment. Table 1 describes the simulation setting in detail.

For the evaluation, the performance of the proposed MCMAC protocol was compared with the MC-LMAC protocol [17], the slotted multi-channel approach especially designed for WSNs, which is known for showing the best performance among the same category of protocols. We compared the two protocols using different performance metrics: packet reception ratio, network throughput, and energy efficiency.

The proposed protocol is designed to gather data from sensor nodes and send them to the sink in a tree-based multi-hop network. Therefore, the tree-based routing scheme (presented in Section 3.2) was used to build the tree topology, and each node has to maintain a child–parent relationship for data transmission. Note that the tree-based routing scheme was applied to both MCMAC and MC-LMAC protocols. Furthermore, it is necessary to determine the length of a cycle time (or the size of a superframe) required for all nodes to send data to the sink. With MCMAC, the sink can determine the size of a superframe based on the bandwidth demand of the network. On the other hand, we cannot determine the superframe size of MC-LMAC exactly, but we can estimate it approximately. However, this may cause some problems. In fact, if the superframe size is too small, some nodes cannot get a free time slot for transmission; otherwise, if the size of a superframe is larger than required, this causes higher latency and lower throughput. Therefore, for fair evaluation, the two protocols were run with the same size of superframe. We first ran the simulations with MCMAC to obtain the value of the superframe size, and then used that value to run the simulations with MC-LMAC.

**Figure 9:**
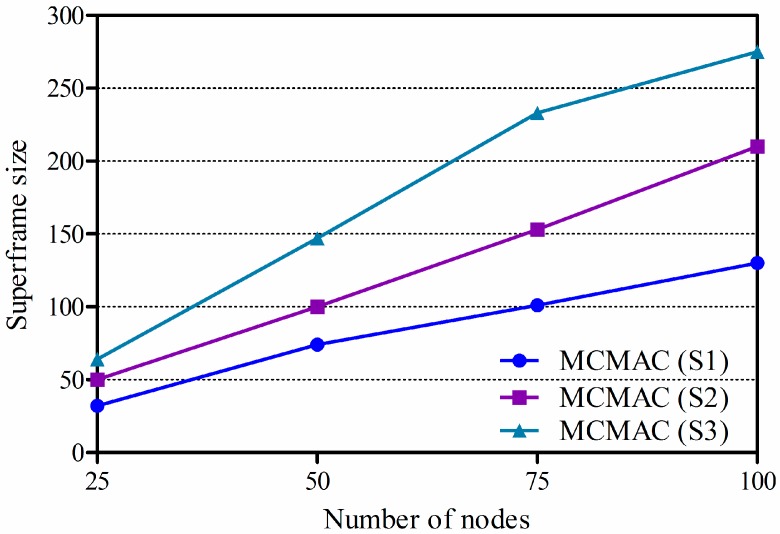
The size of a superframe in MCMAC according to the number of nodes (with η(*.*)=1).

Figure 9 shows the simulation results of the size of a superframe (SF) in MCMAC by changing the number of nodes and the length of the tree topology (by changing the location of the sink in the network). We can see that the SF size increases according to the increasing of the number of nodes and the network length. It should be noted that in MCMAC, the size of a superframe is based on the sharable slot size which is calculated according to the bandwidth demand of each sensor node (as shown in Equation (13)). By using the results of a superframe size in MCMAC, we can run the experiments with MC-LMAC and get the corresponding results for evaluations.

### 4.2. Simulation Results and Discussions

In this section, we discuss the evaluation results of our approach compared to MC-LMAC in terms of packet reception ratio, network throughput, and energy efficiency. All results were obtained through extensive simulations using the QualNet simulator. 

#### 4.2.1. Packet Reception Ratio

In this experiment, it was assumed that every sensor node senses data and transmits only one data packet to the sink in one round of data collection. To evaluate the performance of the two protocols in terms of reliability of data transmission, we use packet reception ratio (*PRR*), which is defined as the ratio of the number of data packets received by the sink to the number of data packets generated by all sensor nodes. Note that *PRR* is measured based on the number of sensor nodes, whether a node sends data or not. In the experiments, the number of nodes changes from 50 to 100, and the number of channels changes from 1 to 8 in the ISM 2.4 GHz band.

**Figure 10:**
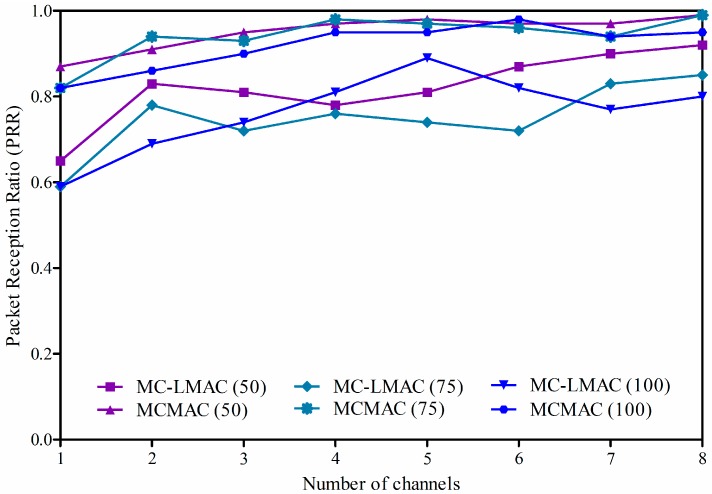
Packet reception ratio according to the number of channels and network density.

Figure 10 shows the *PRRs* of the two protocols according to variation in network density (*i.e.*, changing the number of nodes) and the number of channels to be used. In the figure, the x-axis indicates the number of available channels, whereas the y-axis presents the results of *PRR*. Different graphs show the *PRRs* of the two protocols with different numbers of nodes. The hop count mostly falls within eight hops, according to the deployment area and network density. It is obvious that *PRR* increases in accordance with the increased number of channels for the two protocols, since collision is reduced when the nodes transmit data on different channels in MCMAC, or when a node has more chances to get an interference-free slot in MC-LMAC. It is shown that our proposed protocol, MCMAC, is superior to MC-LMAC in terms of *PRR* in all cases. More specifically, MCMAC achieves more than 95% *PRR*, on average. When the number of channels is small, collisions can occur since the neighboring nodes at the same depth may choose the same channel to receive data. As the number of channels increases, *PRR* also increases, since the neighboring nodes can transmit on different channels. Moreover, as the number of nodes increases, *PRR* is reduced, since nodes with the same parent share the same slot on the same channel. On the other hand, MC-LMAC only achieved more than 80% *PRR* on average. When the number of channels is small, MC-LMAC shows low performance since some of the nodes may not get an interference-free slot. With a network of 100 nodes, a utilized superframe may not be enough for all nodes to get a free slot. When the number of channels increases, more nodes can get control of a time slot, and *PRR* increases accordingly.

Compared to our protocol, MC-LMAC achieves lower *PRR* since it cannot guarantee the reliability of data transmission. In fact, before transmitting data, a sender notifies a receiver of upcoming data, and on which channel to receive data, by sending a preamble during the common frequency (CF) phase within its controlled slot. So, if a receiver does not get a preamble, it cannot get upcoming data from the sender. One more problem that lowers *PRR* in MC-LMAC is the clash when a receiver is addressed by multiple senders at the same time.

#### 4.2.2. Network Throughput

One of the important goals in designing a MAC protocol is to provide better performance in data transmission. Therefore, in this part, we evaluate the two protocols in terms of network throughput, the rate of successful data packets (measured in *bytes*) received at the sink per second, taking into account the impact of network density and the number of channels to be used.

**Figure 11:**
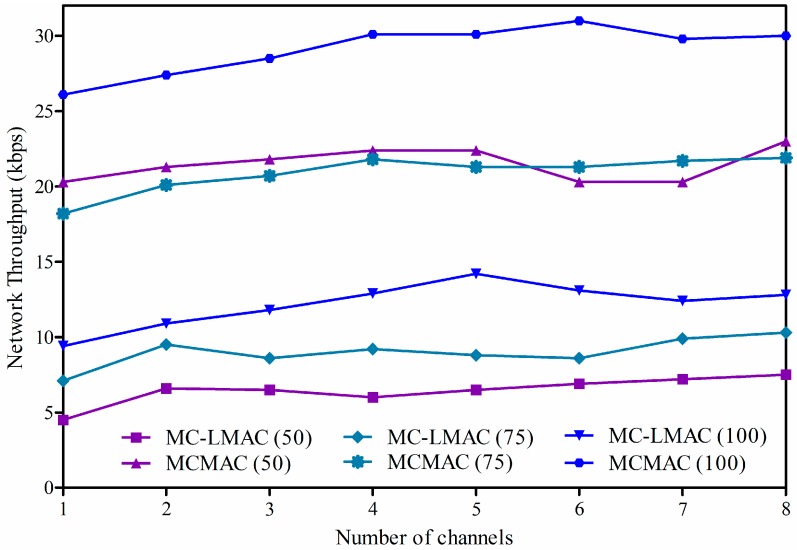
Network throughput according to the number of channels and network density.

The network throughput of the two protocols is presented in Figure 11, where the x-axis indicates the number of available channels to be used, and the y-axis indicates network throughput (in kilobits per second) at the sink. Different graphs present network throughput from changing the number of source nodes. The hop count for different random deployments falls to about eight hops, depending on network density.

As shown in Figure 11, network throughput increases when increasing the number of channels or network density under both protocols. We can see that MCMAC is superior to MC-LMAC in terms of network throughput by more than 50%. On average, MC-LMAC achieves maximum throughput of 14 kbps, whereas our MCMAC protocol can achieve maximum throughput of 30 kbps. In MCMAC, when the number of channels increases, collisions are reduced significantly, since a node can use different channels for data transmission and thus the size of a superframe is effectively reduced by reinforcing parallel transmission. Therefore, with a sufficient number of channels, MCMAC can achieve high performance in network throughput and optimal data collection time. Note that a node always has sufficient time to send data in its buffer, since the slot time is assigned according to the node’s bandwidth demand. On the other hand, in MC-LMAC, a node may not get a free slot for transmission if the number of time slots and channels is not sufficient. Moreover, when a receiver is addressed by multiple senders simultaneously (the clash problem), data can be lost and network throughput reduced significantly.

#### 4.2.3. Energy Efficiency

In a sensor node, the radio consumes the most energy, compared with the other components. Moreover, the energy consumption of a radio when operating in sending, receiving, or listening states is much more than that in idle or sleep mode. For instance, the CC2420 radio, used in TelosB or Kmotes, consumes 23 mA when receiving or listening, 8.5 mA in transmitting at −25 dBm, 21 µA in an idle state, and 1 µA in sleep state [39]. Thus, for energy conservation, the radio should be turned off when it is not required.

Since the energy of a sensor node is constrained, a well-designed approach with effective control of the radio should be able to achieve high energy efficiency to prolong network lifetime. Thus, we use, as the definition of energy efficiency, *consumed energy per successfully received packet* to evaluate how effectively a protocol uses energy for packet transmission. Energy efficiency can be calculated by measuring total energy consumed by all sensor nodes (except for the sink) and then dividing it by the total number of packets received at the sink.

**Figure 12:**
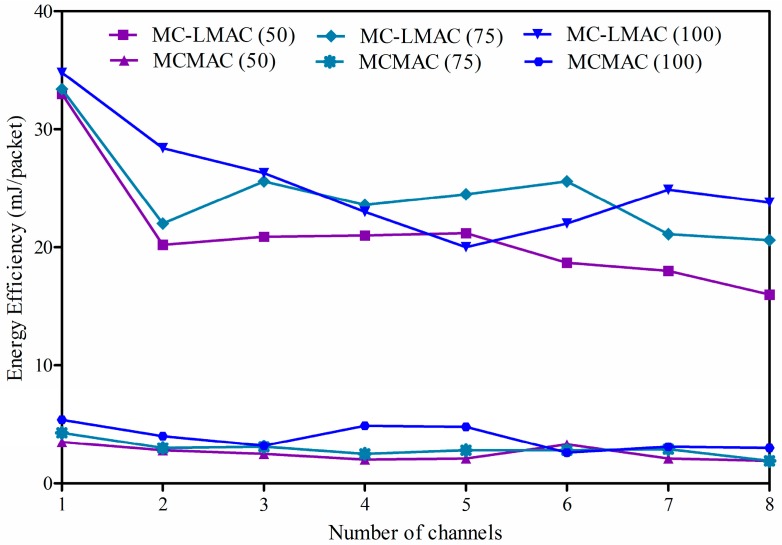
Energy consumption per successfully received packet at the sink.

Energy efficiencies for the two protocols are presented in Figure 12, where the x-axis illustrates the number of available channels and the y-axis presents energy efficiency, expressed in milli-joules per packet. It is shown that the MCMAC protocol significantly outperforms the MC-LMAC protocol. The improvement under MCMAC comes from two aspects: (*i*) a node only wakes up at the scheduled slot to receive or transmit data and goes into sleep mode at other times; (*ii*) packet aggregation and filtering is maximized thanks to the principle design of the proposed slot scheduling, such that a sending slot always comes right after a receiving slot. But a node under MC-LMAC does not know when potential packets will arrive, and thus, it has to remain active during the CF period in every slot. Since the number of CF slots in the CF period is equal to the number of utilized channels, every node has to consume more time waiting for incoming packets when increasing the number of used channels, thereby resulting in higher energy consumption. 

## 5. Conclusions and Future Works

In this work, we propose optimized sharable slot scheduling that aims at optimizing the size of a sharable slot with the use of multiple channels. It also handles general situations where a node can generate multiple packets. Thanks to the improved throughput, the proposed algorithm enables a protocol to support more real-time applications with tighter time constraints for data gathering. According to extensive simulation results, our proposed approach outperforms the MC-LMAC protocol in terms of packet reception ratio, network throughput, and energy efficiency. Therefore, it can be said that our proposed approach is highly suitable for real-time applications in industrial sensor networks.

In fact, the performance of a WSN protocol is significantly affected by the environment. Therefore, the performance of the proposed approach should be verified in the future under different environmental conditions, especially in an industrial environment with a real sensor test-bed. Moreover, a case study for testing the protocol’s performance in handling multimedia data or bursty data should be conducted.

## Figures and Tables

**Table 1:** Some key simulation parameters and values.

Parameter	Value
Number of nodes (*nNodes*)	50−100
Payload length	32 bytes
Noise factor	10 dB
Path loss model	Two-ray
Shadowing model	Constant
Fading model	Rician
Sensor energy model	MicaZ
Transmission power	−25 dBm
Dimensions	100 × 100 m^2^
Simulation time	600 s
Number of frequencies	1–8
MAC protocols	MC-LMAC, MCMAC
Routing protocol	Tree-based routing
Node placement	Random
